# Body size perception in stroke patients with paresis

**DOI:** 10.1371/journal.pone.0252596

**Published:** 2021-06-04

**Authors:** Azam Shahvaroughi-Farahani, Sally A. Linkenauger, Betty J. Mohler, Simone C. Behrens, Katrin E. Giel, Hans-Otto Karnath

**Affiliations:** 1 Division of Neuropsychology, Center of Neurology, Hertie-Institute for Clinical Brain Research, University of Tübingen, Tübingen, Germany; 2 Department of Psychology, Lancaster University, Lancaster, United Kingdom; 3 Max Planck Institute for Biological Cybernetics, Tübingen, Germany; 4 Department of Psychosomatic Medicine and Psychotherapy, University of Tübingen, Tübingen, Germany; Washington University in Saint Louis School of Medicine, UNITED STATES

## Abstract

Recent studies have suggested that people’s intent and ability to act also can influence their perception of their bodies’ peripersonal space. Vice versa one could assume that the inability to reach toward and grasp an object might have an impact on the subject’s perception of reaching distance. Here we tested this prediction by investigating body size and action capability perception of neurological patients suffering from arm paresis after stroke, comparing 32 right-brain-damaged patients (13 with left-sided arm paresis without additional spatial neglect, 10 with left-sided arm paresis and additional spatial neglect, 9 patients had neither arm paresis nor neglect) and 27 healthy controls. Nineteen of the group of right hemisphere stroke patients could be re-examined about five months after initial injury. Arm length was estimated in three different methodological approaches: explicit visual, explicit tactile/proprioceptive, and implicit reaching. Results fulfilled the working hypothesis. Patients with an arm paresis indeed perceived their bodies differently. We found a transient overestimation of the length of the contralesional, paretic arm after stroke. Body size and action capability perception for the extremities thus indeed seem to be tightly linked in humans.

## Introduction

Perception and action are closely intertwined processes. Traditionally, the main purpose of visual perception was seen to assist in the organisation of action [e.g., 1, 2]. However, recent studies have found that people’s intent and ability to act also can influence their perception of (peripersonal) space. For example, it has been observed that holding a tool influences judged distance to the target [3, 4]. In fact, holding the tool influenced perceived distance only if subjects intended to reach the target with the tool. If they simply held the tool but had no intention to reach, the targets were judged the same distance as if they did not hold the tool. In line with the findings by Witt and colleagues, Sposito et al. [5] and Romano et al. [6] reported that tool-use influences the representation of body metrics if the tool became essential to successfully execute a motor task in far space. In the same vein, studies with stroke patients and neglect have shown that using a tool can cause an augmentation of peripersonal space, while others have found a reduction [7–9].

Further examples for an influence of motor acts on visual perception are, e.g., that batting execution prompts judgments of ball size [10] or that returns in tennis prompts judgments about the height of the net [11]. Moreover, Linkenauger et al. [12] demonstrated that right-handed participants judged their right arm as longer than their left arm, and therefore they falsely believed that they could reach objects farther away with their right arm. In contrast, left-handed participants perceived length of both arms accurately. Right-handed subjects also were observed to believe that they could grasp relatively larger objects with their right hands, because they perceived their right hand larger than their left [13].

The majority of the above studies thus suggest that the body and its capabilities scale perceived peripersonal space. Vice versa one could assume that the inability to reach toward and grasp an object, e.g. after loss of full capacity to move one arm after stroke, should have an impact on the subject’s perception of reaching distance. Here we test this prediction by investigating body size and action capability perception of neurological patients suffering from arm paresis after stroke.

While the physiological effects of paresis are well understood, the perceptual effects of paresis are less clear. We know that patients suffering from stroke and contralesional paresis of arm and/or leg usually also experience sensory impairment at the paretic limb(s). The degree of sensory impairment is related to the severity of motor paralysis [14]. Beyond, it has been suggested that stroke patients may have an impairment in own body size perception, e.g. arm length [15, 16]. Vice versa, it was observed that training by using a mirror box may positively affect body representation in patients suffering from motor impairment [17]. Based on these observations, it has been hypothesized that patients with an arm paresis should perceive their bodies differently, namely show a bias in arm length perception. They were also expected to exhibit impaired perception of their action capabilities, i.e. judging how far they can reach. If so, measuring arm size perception and perception of reaching capability might also be a useful diagnostic criterion in the clinical management of these patients. To investigate this hypothesis on the effect of paresis on body perception it was important to control for possible other factors that also can influence (body) perception after stroke, namely primary visual field defects, spatial neglect, and anosognosia.

## Methods

### Participants

**Fig 1:**
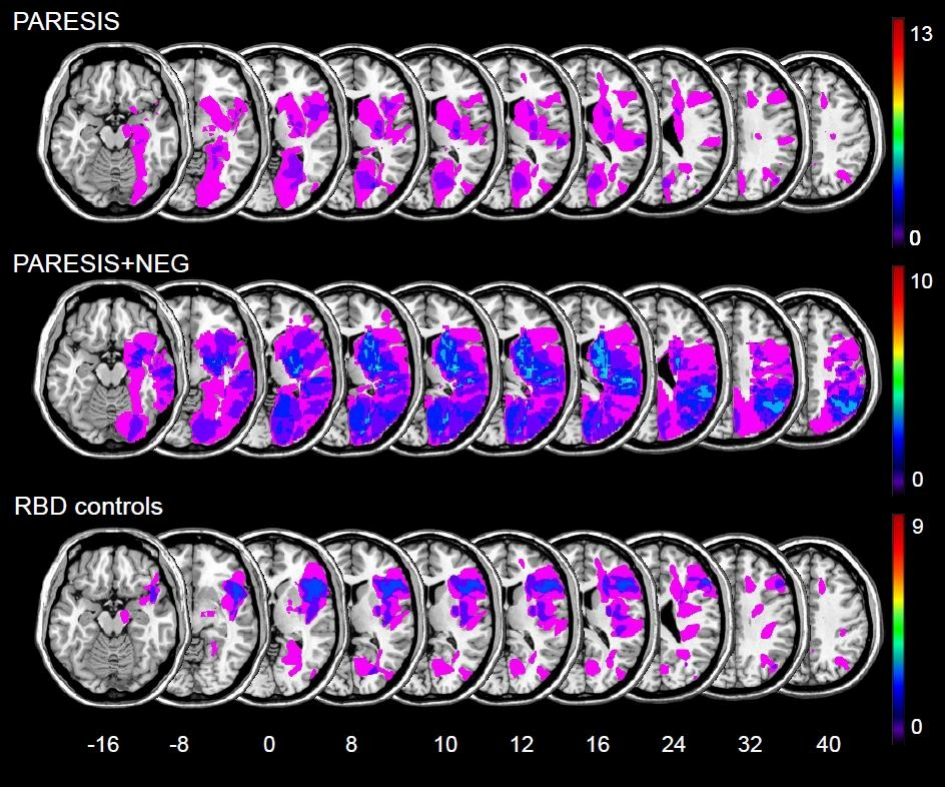
Simple lesion overlay plots of the three brain damaged subject groups (PARESIS, arm paresis without additional spatial neglect or anosognosia; PARESIS+NEG, arm paresis and additional spatial neglect but no anosognosia; RBD controls, stroke patients without arm paresis, without neglect and without anosognosia). The lesion maps are superimposed on the single-subject T1 MNI152 template. The figure shows the vertical z coordinate for each slice of standardized MNI space. For each voxel, the number of patients with a lesion at that location is color coded (n = 1 to max.).

**Fig 2:**
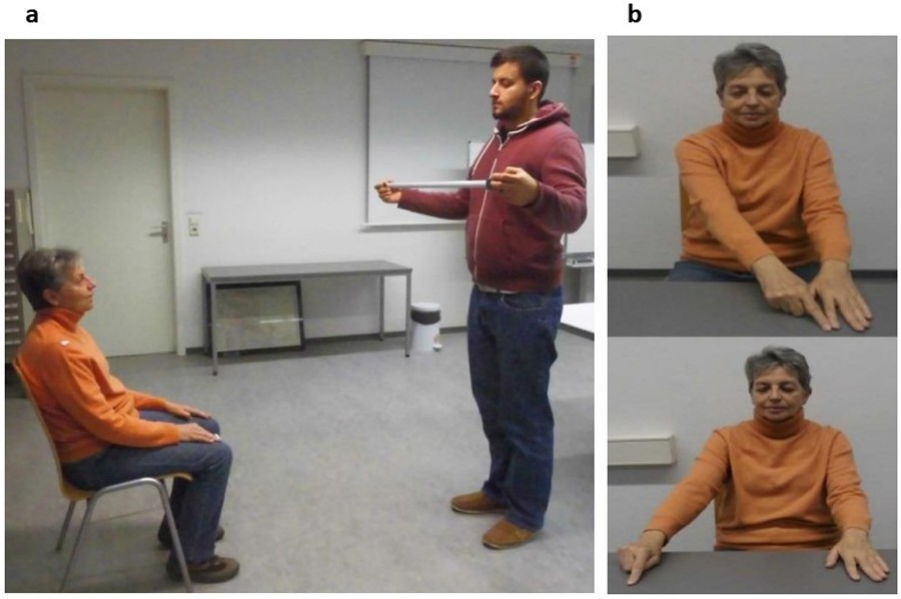
*(****a****) Experiment 1 (Arm length estimate−Visual approach)*: Participants estimate the length of their right/left arm. Arm (plus hand) length was estimated by the participant by having the experimenter adjust a horizontally oriented, retractable tape measure, so that the tape matched the perceived length of the participant’s arm (plus hand). The length of the tape was increased/decreased until its length matched the participant’s perception of his/her arm (plus hand) length. *(****b****) Experiment 2 (Arm length estimate−Tactile/proprioceptive approach)*: Participants seated with eyes closed while estimating the length of their right/left arm (plus hand) on the table by using a tactile starting point. The participant then was instructed to move the right index finger on the table towards the right to indicate the length of his/her right/left arm (plus hand).

Over a period of 1.5 years, thirty-two consecutively admitted patients with first ever right hemisphere stroke hospitalized at the Centre of Neurology at the Tübingen University were recruited for the present study. Exclusion criteria were: diffuse or bilateral brain lesions, acute presence or medical history of other illnesses that affect the central nervous system, evidence of psychiatric episodes in the medical history, evidence of clinically relevant cognitive impairments, non-correctable visual impairments, and presence of anosognosia for hemiparesis. Patients performed clinical and experimental testing on average 1.9 days post-stroke (SD 0.8). Thirteen of them suffered from a left-sided arm paresis without additional spatial neglect (PARESIS), ten showed left-sided arm paresis and additional spatial neglect (PARESIS+NEG), and 9 patients had no arm paresis and no neglect (right brain damaged controls, RBD). All of the patients turned out to be right-handed. Additionally, twenty-seven age-matched healthy right handed participants (non-brain damaged controls, NBD) without neurological or psychiatric disorders were tested. All 59 subjects gave their written informed consent to participate in the study, which was performed in accordance with the ethical standard of the Declaration of Helsinki as revised in 2013 and was approved by the local Ethics Committee of the University of Tübingen (reference no. 172014BO2). Beyond, the individuals pictured in Fig 2 have provided written informed consent (as outlined in PLOS consent form) to publish their image alongside the manuscript. Demographic and clinical data of all subjects are presented in Table 1; simple lesion overlap maps of the patients are illustrated in Fig 1.

**Table 1:** Demographic and clinical data of all 59 participants.

	*PARESIS*	*PARESIS +NEG*	*RBD Controls*	*Healthy Controls*
**Number**	13	10	9	27
**Sex(m/f)**	9/4	7/3	6/3	8/19
**Age(years)**	61.2 ± 12.4	67.8 ± 14.0	63.0 ± 8.2	65.0 ± 6.2
**MMSE**	27.7± 0.8	27.6± 1.1	28.0± 0.9	29.0± 0.8
**Etiology**	11 Inf., 2 Hem.	7 Inf., 3 Hem.	4 Inf., 5 Hem.	----
**Time since lesion (days)**	2.1 ± 1.0	1.7 ± 0.7	1.6 ± 0.7	----
**Visual Field defects (% present)**	0	0	0	----
**Arm paresis (% present)**	100	100	0	----
**BMRC grade**	2.3 ± 1.2	1.6 ± 1.7	5	----
**Spatial neglect scores**				----
**Letter Cancellation(COC)**	0.01 ± 0.003	0.43 ± 0.12	0.02 ± 0.007	----
**Bells Test (COC)**	0.01 ± 0.004	0.44 ± 0.11	0.02 ± 0.005	----
**Copying**	0	4.0 ± 2.8	0	----
**Anosognosia scores**	0	0	0	----

Data are presented as mean ± SD. PARESIS, patients with left-sided arm paresis without additional spatial neglect; PARESIS+NEG, patients with left-sided arm paresis and additional spatial neglect; RBD controls, patients without arm paresis and neglect. MMSE, Mini Mental State Examination [19]. Hemorrhagic (hem); infarct (inf); male (m); female (f). BMRC, British Medical Research Council scale. Center of Cancellation (COC) [23]; copying: the maximum neglect omission score was 8.

### Procedure

After providing informed consent to participate in the study, participants underwent a comprehensive clinical assessment and an experimental session. The whole procedure took 60 minutes. Between clinical assessment and experimental session, participants had a break.

#### Clinical assessment

Handedness was assessed through self-report and by the Edinburgh Handedness Inventory [18]. Clinically relevant cognitive impairments were assessed using the Mini Mental State Examination (MMSE [19]). Arm motor function was quantified based on the commonly used BMRC (British Medical Research Council) scale. The BMRC grades range from zero to five (0: no movement, 1: palpable flicker, 2: movement without gravity, 3: movement against gravity but not against resistance, 4: movement against mild resistance, 5: normal movement). Visual field defects were assessed by the standard neurological confrontation technique. The patients were asked to signal as soon as they perceived the examiner’s waving fingers move inward from beyond the boundaries of each visual field quadrant.

The following clinical neglect tests were applied: Letter Cancellation Task [20], Bells Test [21], and a Copying Task [22]. All three tests were presented on a horizontally oriented 21*29.7 cm sheet of paper. In the Letter Cancellation Task, 60 target letters ‘A’ are distributed amid distracters. Patients were asked to cancel all of the targets. The Bells Test requires identifying 35 bell symbols distributed on a field of other symbols. In the Copying Task, patients were asked to copy a complex multi-object scene consisting of four figures (a fence, a car, a house and a tree), two in each half of the test sheet. For the Letter Cancellation Task and the Bells Test, we calculated the Center of Cancellation (CoC) using the procedure and software by Rorden and Karnath [23]. This measure is sensitive to both the number of omissions and the location of these omissions. CoC scores >.09 in the Letter Cancellation Task and the Bells Test were taken to indicate neglect behavior [cf. 23]. In the Copying Task, omission of at least one of the contralateral features of each figure was scored as 1, and omission of each whole figure was scored as 2. One additional point was given when contralesional figures were drawn on the ipsilesional side of the test sheet. The maximum score was 8. Any score higher than 1 (i.e., >12.5% omissions) indicated spatial neglect [22]. For a firm diagnosis of spatial neglect in the acute stage of the stroke, the patients had to fulfill the above criteria in at least two of the three tests. At the time of the second (chronic) examination, patients were classified as showing chronic neglect when they fulfilled the above criteria in at least one of the three tests.

Anosognosia for arm paresis was examined using the anosognosia scale by Bisiach et al. [24] together with the diagnosis criteria of Baier and Karnath [25]: Patients who spontaneously mention the disorder or who report the disorder following a specific question about the strength of the patient’s limb(s) (Grades 0 or 1) are classified as not showing anosognosia. Patients who do not acknowledge their paresis/plegia even after a specific question about the strength of their arm or after demonstration of the deficit (Grades 2 or 3) are diagnosed as suffering from anosognosia.

### Experimental assessment

#### Experiment 1: Arm length estimate − visual approach

The participant sat opposite of the experimenter, placing the hands on the thighs with eyes open (Fig 2A). Markers were adhered to the shoulder joint (where the clavicle is connected to the humerus) and to the tip of the index finger on both the left and right sides of the body. The participant was initially asked to estimate right arm length, i.e. the length from the marker on the shoulder joint to the fingertip of the extended right hand. Arm (plus hand) length was estimated by the participant by having the experimenter adjust a horizontally oriented, retractable tape measure, so that the tape matched the perceived length of the participant’s arm (plus hand). The participant was asked to say ‘stop’ when he or she perceived that arm length (plus hand) matched tape length. The numbers of the tape measure were intentionally hidden from the participant’s view. Length estimates were performed 10 times. In five trials, the experimenter pulled the retractable tape measure slowly out until the subjective estimate was reached; in five trials the retractable tape measure was extended to about 1.5m and was then slowly shortened until the subjective estimate was reached. Sequence of the 10 trials was randomized. Subsequently, participants estimated the length of the left arm by performing another 10 estimation trials.

#### Experiment 2: Arm length estimate–tactile/proprioceptive approach

Participants were sitting in the middle of a table with eyes closed. The experimenter took the participant’s left hand and put it to a randomized location on the table (Fig 2B). The index finger of the left hand was used as the tactile starting point. Now the experimenter instructed the participant to touch the left index finger with the right index finger. The participant then was instructed to move the right index finger on the table towards the right to indicate the length of their right arm (plus hand). This procedure was repeated 5 times for the subjective estimate of right arm length. Subsequently, subjects estimated the length of their left arm (plus hand) by using exactly the same procedure. The latter was necessary because in the PARESIS and PARESIS+NEG subjects the left arm was paretic/plegic.

#### Experiment 3: Arm length (to grasp) estimate − implicit approach

Participants were sitting at a horizontal table (1.30*0.30 m) with eyes open. The experimenter moved a round target (diameter 2cm) along the midline of the table which was aligned with the subject’s midsagittal trunk plane. In 5 trials, target position started far out of subject’s reach and was slowly moved in straight line towards the subject. The task was to verbally instruct an experimenter to stop at exactly the position that the subject thought “that he/she could just reach and grasp the target by a precision grip without moving the shoulders”. In 5 trials, target position started close to the subject’s trunk and was slowly moved radially until the subject felt “that he/she just could no longer reach and grasp the target by a precision grip without moving the shoulders”. Sequence of the 10 trials was randomized. The procedure was repeated for two further directions on the table (-45°, +45° from the subject’s midsagittal trunk plane). Data were averaged over the three directions. Afterwards, the actual reachability for each arm in each direction was measured. Also, these data were averaged over the three directions.

Subsequent to all three *Experiments 1*, *2*, *& 3* (above), actual lengths of the participants’ both arms were measured between the tip of the acromial process and the tip of the left/right index finger (with extended hand position). On the paretic body side, the arm was passively stretched by the experimenter.

Nineteen of the 32 right hemisphere stroke patients could be re-examined about five months after their initial injury (on average 167.3 days post-stroke [SD 1.6]). Thirteen patients were excluded from subsequent analysis. Reasons for exclusion were as follows: three patients had a second stroke, six patients did not consent to follow-up testing, and four patients had moved far beyond the catchment area. Eight of the 19 re-investigated patients were from the group with left-sided arm paresis without additional spatial neglect or anosognosia (PARESIS), 4 from the group with left-sided arm paresis and additional spatial neglect but no anosognosia (PARESIS+NEG), and 7 patients from the group without arm paresis, without neglect and without anosognosia (right brain damaged controls, RBD).

In Experiments 1 and 2, accuracy ratios were calculated by dividing the estimated length by the subjects’ actual arm length; in Experiment 3, by dividing the estimated reachability-distance by (i) the subjects’ actual, maximal reaching distance or (ii) the subjects’ actual arm length. Accuracy ratios over 1 thus signaled overestimation of arm length; accuracy ratios under 1 underestimation of arm length. For statistical analyses of the accuracy ratios in the three experiments depending on the equality of variances, one-way ANOVAs or Welch tests were conducted under SPSS (SPSS Inc., Vers. 22) separately for each ‘body side’ (left arm, right arm) using the between-subject factor ‘group’ (PARESIS, PARESIS+NEG, RBD, NBD).

## Results

### Experiment 1: Arm length estimate − visual approach

**Fig 3:**
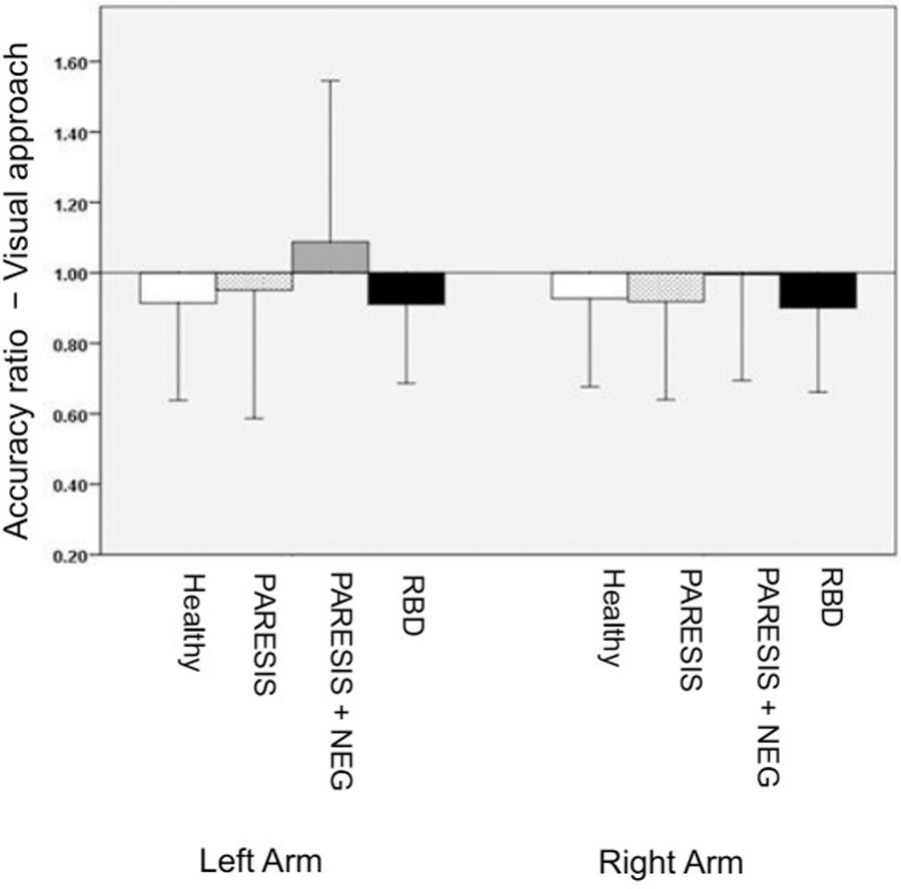
Experiment 1 (arm length estimate − visual approach): Mean accuracy ratios of the four subject groups measured in the acute phase of the stroke. Bars represent standard deviations. Healthy, non-brain damaged controls without neurological or psychiatric disorders; PARESIS, arm paresis without additional spatial neglect or anosognosia; PARESIS+NEG, arm paresis and additional spatial neglect but no anosognosia; RBD, right brain damaged control patients without arm paresis, neglect or anosognosia.

Accuracy ratios of the four subject groups are illustrated in Fig 3. Levene’s test revealed comparable variances for the right body side (F (3,55) = 0.512, p = 0.67) and variance differences for the left (F (3,55) = 2.786, p = 0.049). Statistical analysis revealed that accuracy ratios did not differ significantly between groups neither for the right body side (ANOVA: F (3, 55) = 1.004, p = .398), nor for the left body side (Welch’s test: F (3, 21.405) = 1.754, p = .186).

### Experiment 2: Arm length estimate–tactile/proprioceptive approach

**Fig 4:**
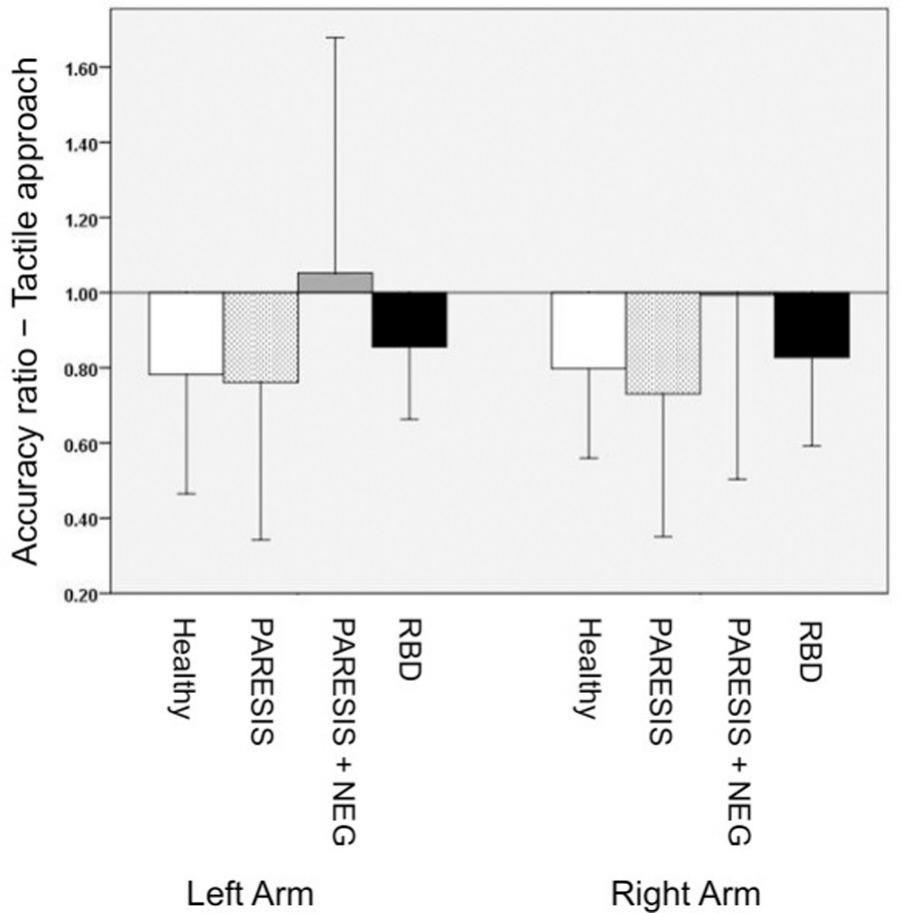
Experiment 2 (arm length estimate–tactile/proprioceptive approach): Mean accuracy ratios of the four subject groups. Bars represent standard deviations. Healthy, PARESIS, PARESIS+NEG, RBD as in Fig 3.

Accuracy ratios of the four subject groups are illustrated in Fig 4. Levene’s test revealed variance differences for both the right body side (F (3,40) = 3.099, p = 0.037) as well as the left body side (F (3,40) = 5.645, p = 0.003). Statistical analysis revealed no significant difference in arm length estimation scores between the groups neither for the right body side (Welch’s test: F (3, 21.128) = 2.71, p = .071), nor the left body side (Welch’s test: F (3, 21.188) = 2.565, p = .082).

### Experiment 3: Arm length estimate (to grasp) − implicit approach

**Fig 5:**
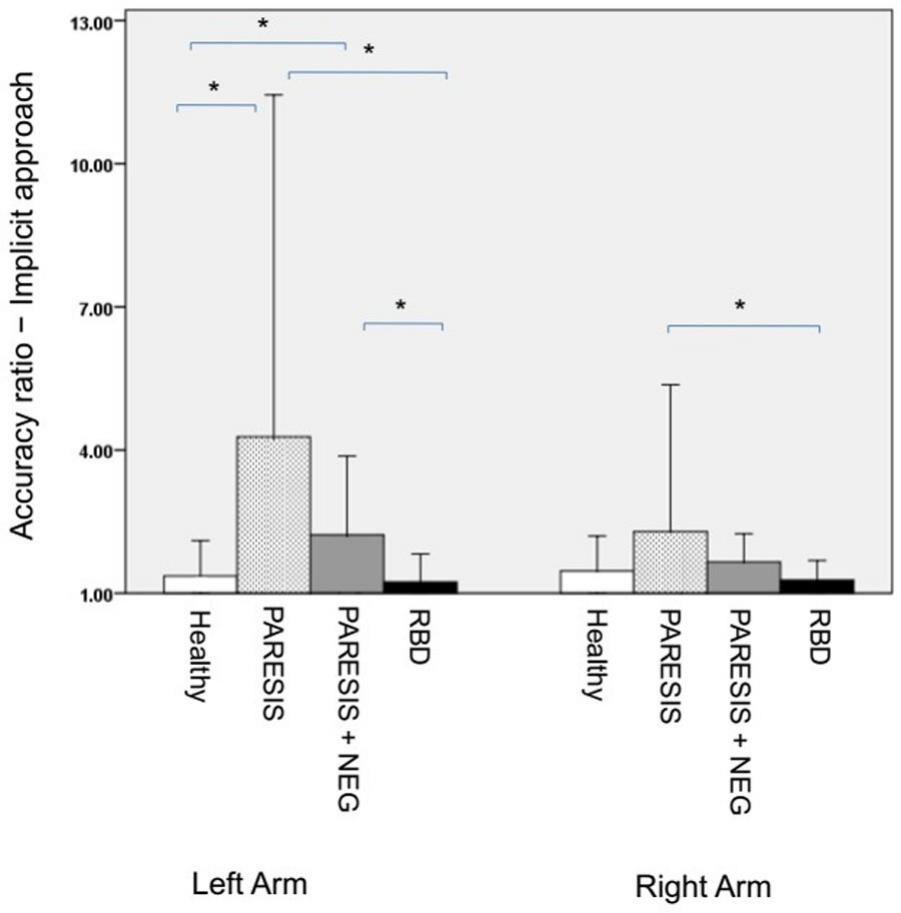
Experiment 3 (arm length estimate *(to grasp)* − implicit approach): Mean accuracy ratios of the four subject groups by dividing the estimated reachability-distance by the subjects’ actual, maximal reaching distance. Note that six paretic patients had to be excluded for this analysis since they were not able to move the contralesional arm and actual reachability thus could not be measured. Bars represent standard deviations. Healthy, PARESIS, PARESIS+NEG, RBD as in Fig 3. *, significant difference.

We had to exclude six patients with hemiparesis/-plegia (3 with neglect and 3 without neglect) from the analysis. They were not able to move the paretic arm; actual reachability thus could not be measured. Accuracy ratios of the remaining participants are illustrated for the four subject groups in Fig 5. Levene’s test revealed variance differences for both the right body side (F (3,49) = 4.271, p = 0.009) and left body side (F (3,49) = 9.914, *p* < .001). Welch’s tests, separately for each body side, revealed that accuracy ratios differed significantly between groups for both sides (right body side: F (3, 17.73) = 3.89, p = .027; left body side: F (3, 15.292) = 4.881, p = .014). For the right body side, pairwise post-hoc Dunn tests with Bonferroni-Holm-correction revealed only one significant difference between paretic patients without neglect vs. right brain damaged controls (p = .002). For the left body side, pairwise post-hoc Dunn test with Bonferroni-Holm-correction were significant for paretic patients without neglect vs. healthy controls (p < .001), paretic patients with neglect vs. healthy controls (p = 0 .006), paretic patients without neglect vs. right brain damaged controls (p < .001), and paretic patients with neglect vs. right brain damaged controls (p = .006).

**Fig 6:**
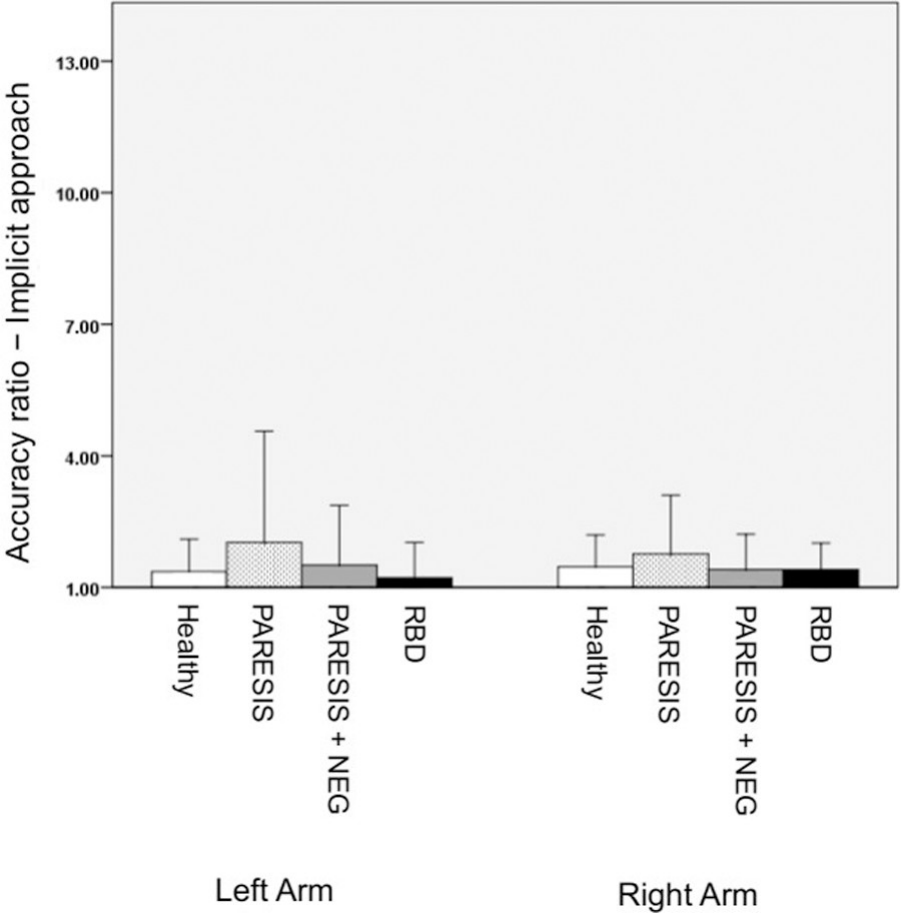
Experiment 3 (arm length estimate *(to grasp)* − implicit approach): Mean accuracy ratios of nineteen patients from the four subject groups measured in the chronic phase of the stroke as well as the mean accuracy ratio from healthy controls. Bars represent standard deviations. Healthy, PARESIS, PARESIS+NEG, RBD as in Fig 3.

At the time point of re-examination about 5 months after initial injury, the grade of arm paresis in all hemiparetic patients (with and without neglect) had improved (acute phase: M = 1.6 (SD 1.2); chronic phase: M = 2.2 (SD 0.8); Paired T-test (11) = 2.548, p = 0.027). Likewise, in all 4 re-examined patients from the PARESIS+NEG group severity of spatial neglect had improved but was still present (acute phase [CoC averaged across letter and bells tests]: M = 0.51 (SD 0.22); chronic phase: M = 0.34 (SD 0.16); Paired T-test (3) = 4.348, p = 0.022). For statistical testing, the same twenty-seven age-matched healthy participants right-handed (non-brain damaged controls, NBD) without neurological or psychiatric disorders were included. Accuracy ratios of the NBD group and the three patient groups in the chronic phase of the stroke are illustrated in Fig 6. Levene’s test revealed variance differences for the right body side (F (3, 45) = 3.088, p = 0.036) as well as for the left body side (F (3, 45) = 12.278, p < .001). Welch’s tests, separately for each body side, showed that accuracy ratios did not differ significantly between groups (right body side: F (3, 10.512) = .703, p = .571; left body side: F (3, 9.72) = 1.4, p = .301).

## Discussion

The present study aimed to investigate body size and action capability perception for the extremities of neurological patients suffering from arm paresis after stroke. If patients had to implicitly estimate the lengths of their arms by asking them about reachability of a visual target, we observed that paretic patients with and without neglect significantly overestimated contralesional left arm length in contrast to healthy controls as well as to RBD patients without paresis. This effect seemed to be related to the paresis of the contralesional arm per se and not to additional neglect: the comparison between accuracy ratios for paretic patients with neglect versus without neglect were not significantly different. The overestimation of contralesional arm length in these groups appeared to be transient: when we reexamined part of the patient sample about five months after their initial injury, this overestimation of left arm length was no longer apparent.

We also observed significantly higher accuracy ratios for the ipsilesional right body side for paretic patients without neglect than for right brain damaged patients without paresis. However, this bias of right arm length perception should be interpreted with great caution. Neither have we found a significant difference of right arm length perception of this patient group from healthy controls, nor have we observed that the second patient group suffering from paresis (PARESIS+NEGLECT) showed such misperception of ipsilesional arm length.

Our results appear to be in line with previous observations in neurological patients suffering from paresis. Fuentes and colleagues [26] investigated patients with hemiplegia due to spinal cord injury. They found striking distortions in the body image of healthy adults, with a large and systematic overestimation of width relative to height when testing participants’ implicit perceived size of body parts and overall body configuration. Interestingly, this widening distortion was found to be reduced in paraplegic and tetraplegic patients who perceived their torso and limbs as elongated relative to their body width. Moreover, Nuara and colleagues [27] reported altered body self-representation in children with unilateral cerebral palsy. Such patients portrayed their upper limbs more asymmetrically relative to controls.

Our results clearly pointed out that overestimation of arm length was related to the paresis per se and not to additional neglect. However, it has been observed in previous work that spatial neglect also has an effect on body representation, although this is not an overestimation of the length of own extremities. Several studies have suggested specific body representation deficits in neglect patients. For example, Coslett [28] observed that patients affected by neglect failed to identify images of left hands but not images of right hands when performing a hand-laterality task. He suggested that neglect may be associated with a disruption of, or failure to attend to, the body schema. Baas et al. [29] used experimental tasks in which photographs of left and right hands as well as left and right rear-view mirrors presented from the front and the back had to be judged as left or right. Their findings suggested that deficient body representation is indeed the major mechanism underlying neglect. In line with this conclusion, Di Vita et al. [30] found that stroke patients with neglect have a deficit in creating an efficient topological map of the body based on a pervasive body representation disorder.

Moreover, also without any brain damage altered perception of body representation has been observed in individuals with peripheral neurological disorders. For example, in case of missing sensory input, as in amputated body parts, the phantom body part was initially experienced as having normal length and size but was increasingly perceived as becoming telescoped (i.e., shortened in length) and small until only a body part remained, dangling from the limb [31]. Other investigations indicated that pain and numbing may influence perceived body size. Patients with complex regional pain syndrome (CRPS) were reported to perceive the affected limb to be larger than it actual was [32, 33]. It was speculated that this distortion of body image could be a critical part of the presentation of CRPS [32]. To add to this, loss of peripheral information by amputee patients concerning the lower right limb impacted the ability to represent the position and relationships among various body parts when attempting to assemble tiles representing these body parts upon a wooden board [34].

Not only in neurological patients but in healthy individuals too, it has been observed that they may have a distorted view of the relative size of their body parts. Linkenauger and colleagues [12] showed that right-handed participants judged their right arm as longer than their left arm, though there was actually no difference in length between the two. In contrast, left-handed participants perceived both arms accurately. Fuentes et al. [26] displayed body parts in proper scale (e.g., the head) on a computer screen and asked healthy subjects to indicate the relative location of the remaining body parts. The authors found that the width of their shoulders and the length of their upper arms were overestimated, while the lengths of forearms and lower legs were underestimated. Similarly, more recent studies [35, 36] observed an overall overestimation in healthy participants when judging lengths of body parts via inferring the amount of times a form of metric standard (an object or a body part) would fit into the segment they were asked to evaluate. Thus, a distorted view of the relative size of body parts does not seem to be pathological per se; some distortions of body perception seem to be common to all people. Before drawing conclusions about distorted body perception in clinical populations, the pattern of such ‘normal’ biases must be analyzed and considered.

Therefore, we view the one significant difference in accuracy ratios for the ipsilesional right body side in paretic patients without neglect (see above) with extreme caution. In particular, we did not find a significant difference in right arm length perception of this patient group compared to healthy controls. In contrast, the results of our study are very clear with respect to the contralesional, paretic side of the patients’ bodies. They clearly demonstrated that neurological patients with arm paresis do indeed exhibit a transient overestimation of the length of their paretic arm. Thus, limiting the mobility of an arm indeed seems to have an influence on the perception of the size of the paretic limb.

## References

[1] Gibson JJ. The Perception of the Visual World. Boston: Houghton Mifflin, 1950.

[2] Goodale MA, Milner AD. Separate visual pathways for perception and action. Trends Neurosci. 1992; 15: 20–25. doi: 10.1016/0166-2236(92)90344-8 1374953

[3] Witt JK, Proffitt DR, Epstein W. Tool use affects perceived distance but only when you intend to use it. J Exp Psychol Hum Percept Perform. 2005; 31: 880–888. doi: 10.1037/0096-1523.31.5.880 16262485

[4] Witt JK, Proffitt DR. Action-specific influences on distance perception: A role for motor simulation. J Exp Psychol Hum Percept Perform. 2008; 34: 1479–1492. doi: 10.1037/a0010781 19045987 PMC3490620

[5] Sposito A, Bolognini N, Vallar G, Maravita A. Extension of perceived arm length following tool-use: clues to plasticity of body metrics. Neuropsychologia 2012; 50: 2187–2194. doi: 10.1016/j.neuropsychologia.2012.05.022 22683448

[6] Romano D, Uberti E, Caggiano P, Cocchini G, Maravita A. Different tool training induces specific effects on body metric representation. Exp. Brain Res. 2019; 237: 493–501. doi: 10.1007/s00221-018-5405-1 30460395

[7] Costantini M, Frassinetti F, Maini M, Ambrosini E, Gallese V, Sinigaglia C. When a laser pen becomes a stick: Remapping of space by tool-use observation in hemispatial neglect. Exp Brain Res. 2014; 232: 3233–3241. doi: 10.1007/s00221-014-4012-z 24942702

[8] Pegna AJ, Petit L, Caldara-Schnetzer A-S, Khateb A, Annoni J-M, Sztajzel R, et al. So near yet so far: neglect in far or near space depends on tool use. Ann Neurol. 2001; 50: 820–822. doi: 10.1002/ana.10058 11761484

[9] Berti A, Frassinetti F. When far becomes near: remapping of space by tool use. J Cogn Neurosci. 2000; 12: 415–420. doi: 10.1162/089892900562237 10931768

[10] Witt JK, Proffitt DR. See the ball, hit the ball: apparent ball size is correlated with batting average. Psychol Sci. 2005; 16: 937–938. doi: 10.1111/j.1467-9280.2005.01640.x 16313656

[11] Witt JK, Sugovic M. Performance and ease influence perceived speed. Perception 2010; 39: 1341–1353. doi: 10.1068/p6699 21180356

[12] Linkenauger SA, Witt JK, Stefanucci JK, Bakdash JZ, Proffitt DR. The effects of handedness and reachability on perceived distance. J Exp Psychol Hum Percept Perform. 2009; 35: 1649–1660. doi: 10.1037/a0016875 19968426 PMC3291021

[13] Linkenauger SA, Witt JK, Bakdash JZ, Stefanucci JK, Proffitt DR. Asymmetrical body perception: a possible role for neural body representations. Psychol Sci. 2009; 20: 1373–1380. doi: 10.1111/j.1467-9280.2009.02447.x 19788528 PMC2858772

[14] Umeki N, Murataa J, Kubotaa S, Kogoa H, Yamaguchib T, Higashijimaa M. Relationship between motor paralysis and impairments in tactile sensitivity in elderly stroke patients. Int J Gerontol. 2018; 12: 310–313.

[15] Critchley M. The Parietal Lobes. London: Edward Arnold, 1953.

[16] Ehrsson HH, Kito T, Sadato N, Passingham RE, Naito E. Neural substrate of body size: illusory feeling of shrinking of the waist. PLoS Biol. 2005; 3: e412. doi: 10.1371/journal.pbio.0030412 16336049 PMC1287503

[17] Tosi G, Romano D, Maravita A. Mirror box training in hemiplegic stroke patients affects body representation. Front Hum Neurosci. 2018; 11: 617. doi: 10.3389/fnhum.2017.00617 29354040 PMC5758498

[18] Oldfield RC. The assessment and analysis of handedness: the Edinburgh inventory. Neuropsychologia 1971; 9: 97–113. doi: 10.1016/0028-3932(71)90067-4 5146491

[19] Folstein MF, Folstein SE, McHugh PR. “Mini-mental state”: a practical method for grading the cognitive state of patients for the clinician. J Psychiatr Res. 1975; 12: 189–198. doi: 10.1016/0022-3956(75)90026-6 1202204

[20] Weintraub S, Mesulam M-M. Mental state assessment of young and elderly adults in behavioral neurology. In: Mesulam M-M, editor. Principles of Behavioral Neurology. Philadelphia: F.A. Davis Company, 1985; pp. 71–123.

[21] Gauthier L, Dehaut F, Joanette Y. The bells test: a quantitative and qualitative test for visual neglect. Int J Clin Neuropsychol. 1989; 11: 49–54.

[22] Johannsen L, Karnath H-O. How efficient is a simple copying task to diagnose spatial neglect in its chronic phase? J Clin Exp Neuropsychol. 2004; 26: 251–256. doi: 10.1076/jcen.26.2.251.28085 15202544

[23] Rorden C, Karnath H-O. A simple measure of neglect severity. Neuropsychologia 2010; 48: 2758–2763. doi: 10.1016/j.neuropsychologia.2010.04.018 20433859 PMC3129646

[24] Bisiach E, Vallar G, Perani D, Papagno C, Berti A. Unawareness of disease following lesions of the right hemisphere: anosognosia for hemiplegia and anosognosia for hemianopia. Neuropsychologia 1986; 24: 471–482. doi: 10.1016/0028-3932(86)90092-8 3774133

[25] Baier B, Karnath H-O. Incidence and diagnosis of anosognosia for hemiparesis. J Neurol Neurosurg Psychiatry 2005; 76: 358–361. doi: 10.1136/jnnp.2004.036731 15716526 PMC1739568

[26] Fuentes CT, Pazzaglia M, Long M, Scivoletto G, Haggard P. Body image distortions following spinal cord injury. J Neurol Neurosurg Psychiat. 2013; 84: 201–207. doi: 10.1136/jnnp-2012-304001 23204474

[27] Nuara A, Papangelo P, Avanzini P, Fabbri-Destro M. Body representation in children with unilateral cerebral palsy. Front Psychol. 2019; 10: 354. doi: 10.3389/fpsyg.2019.00354 30837926 PMC6389686

[28] Coslett HB. Evidence for a disturbance of the body schema in neglect. Brain Cogn. 1998; 37: 527–544. doi: 10.1006/brcg.1998.1011 9733563

[29] Baas U, de Haan B, Grässli T, Karnath H-O, Mueri R, Perrig WJ, et al. Personal neglect–A disorder of body representation? Neuropsychologia 2011; 49: 898–905. doi: 10.1016/j.neuropsychologia.2011.01.043 21303677

[30] Di Vita A, Palermo L, Piccardi L, Di Tella J, Propato F, Guariglia C. Body representation alterations in personal but not in extrapersonal neglect patients. Appl Neuropsychol Adult 2017; 24: 308–317. doi: 10.1080/23279095.2016.1174866 27183152

[31] Ramachandran VS, Hirstein W. The perception of phantom limbs. The D. O. Hebb lecture. Brain 1998; 121: 1603–1630. doi: 10.1093/brain/121.9.1603 9762952

[32] Moseley GL. Distorted body image in complex regional pain syndrome. Neurology 2005; 65: 773. doi: 10.1212/01.wnl.0000174515.07205.11 16157921

[33] Peltz E, Seifert F, Lanz S, Müller R, Maihöfner C. Impaired hand size estimation in CRPS. J Pain. 2011; 12: 1095–1101. doi: 10.1016/j.jpain.2011.05.001 21741321

[34] Palermo L, Di Vita A, Piccardi L, Traballesi M, Guariglia C. Bottom-up and top-down processes in body representation: A study of brain-damaged and amputee patients. Neuropsychology 2014; 28: 772–781. doi: 10.1037/neu0000086 24799290

[35] Linkenauger SA, Wong HY, Geuss M, Stefanucci JK, Mc Culloch KC, Bülthoff HH, et al. The perceptual homunculus: the perception of the relative proportions of the human body. J Exp Psychol Gen. 2015; 144: 103–113. doi: 10.1037/xge0000028 25494548

[36] Sadibolova R, Ferrè ER, Linkenauger SA, Longo MR. Distortions of perceived volume and length of body parts. Cortex 2019; 111: 74–86. doi: 10.1016/j.cortex.2018.10.016 30471452

